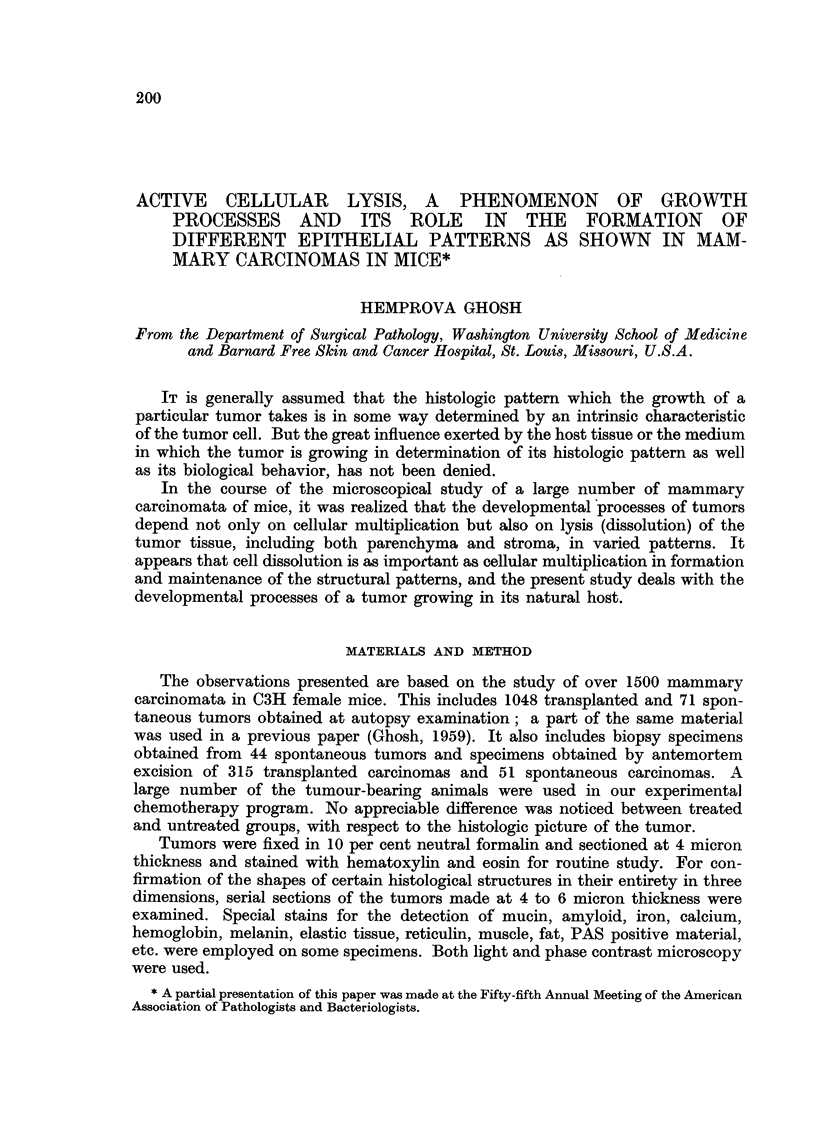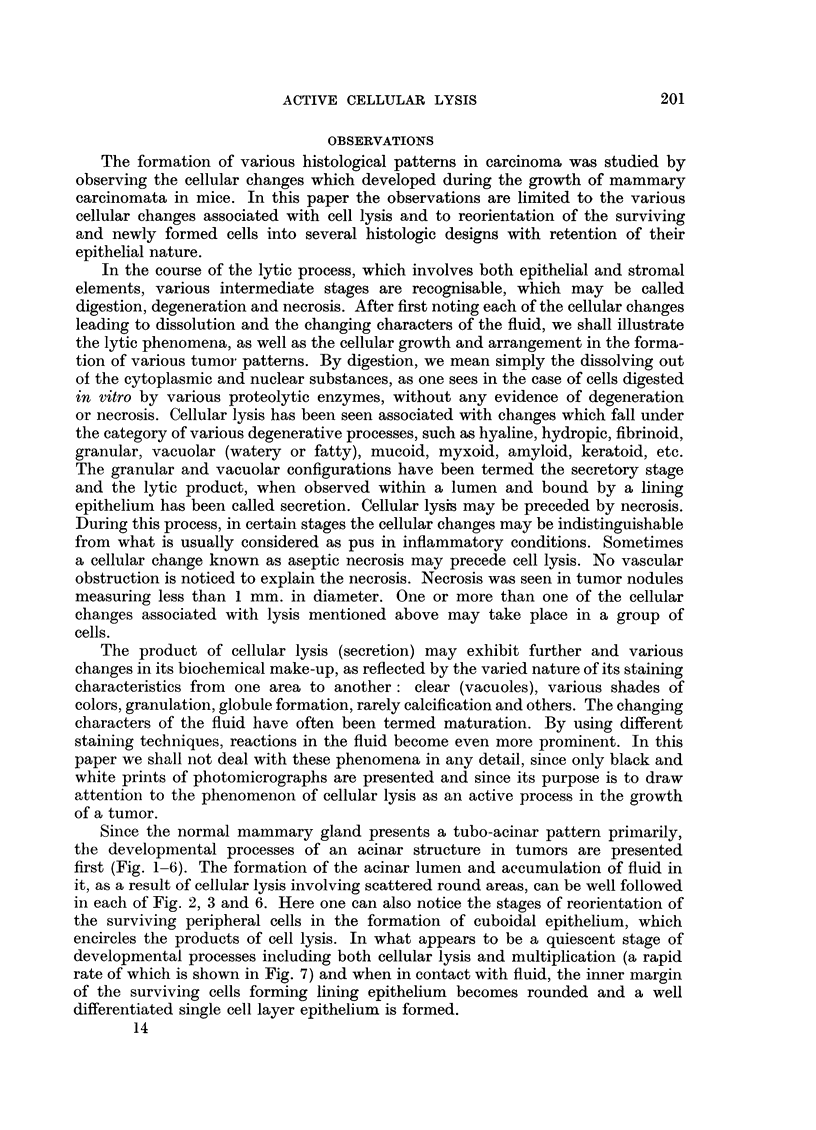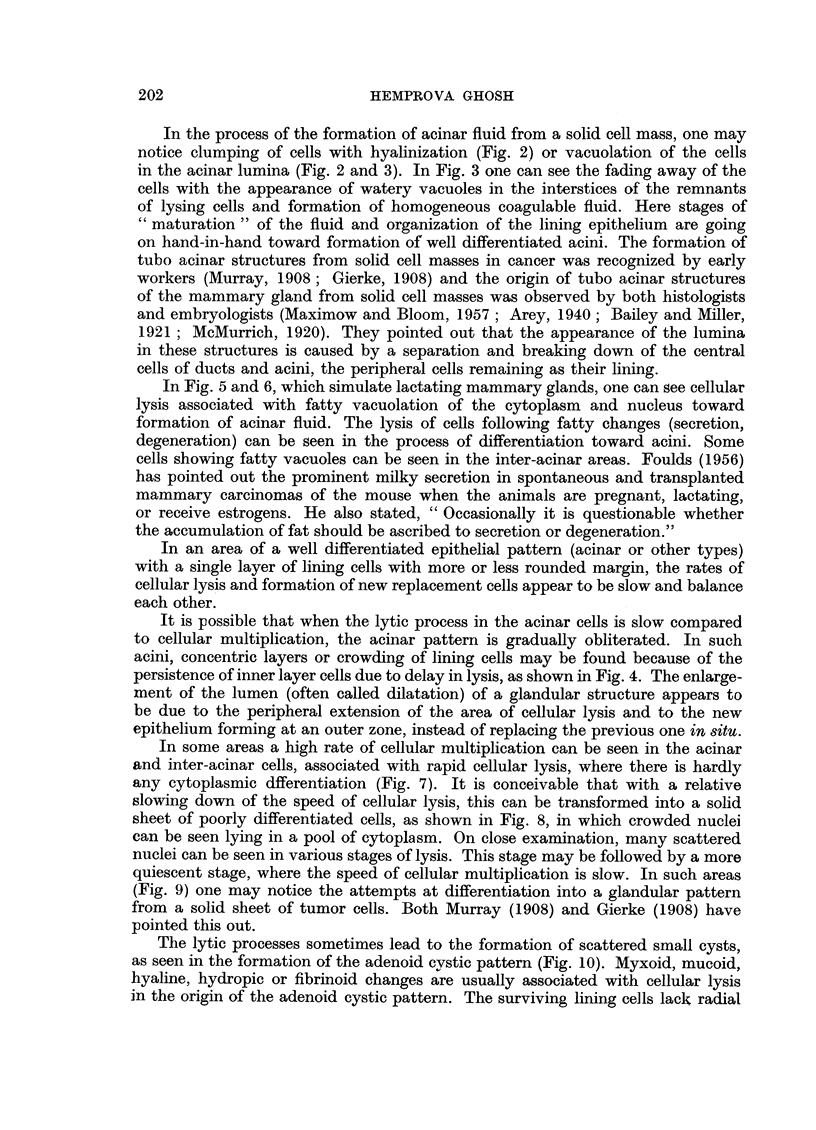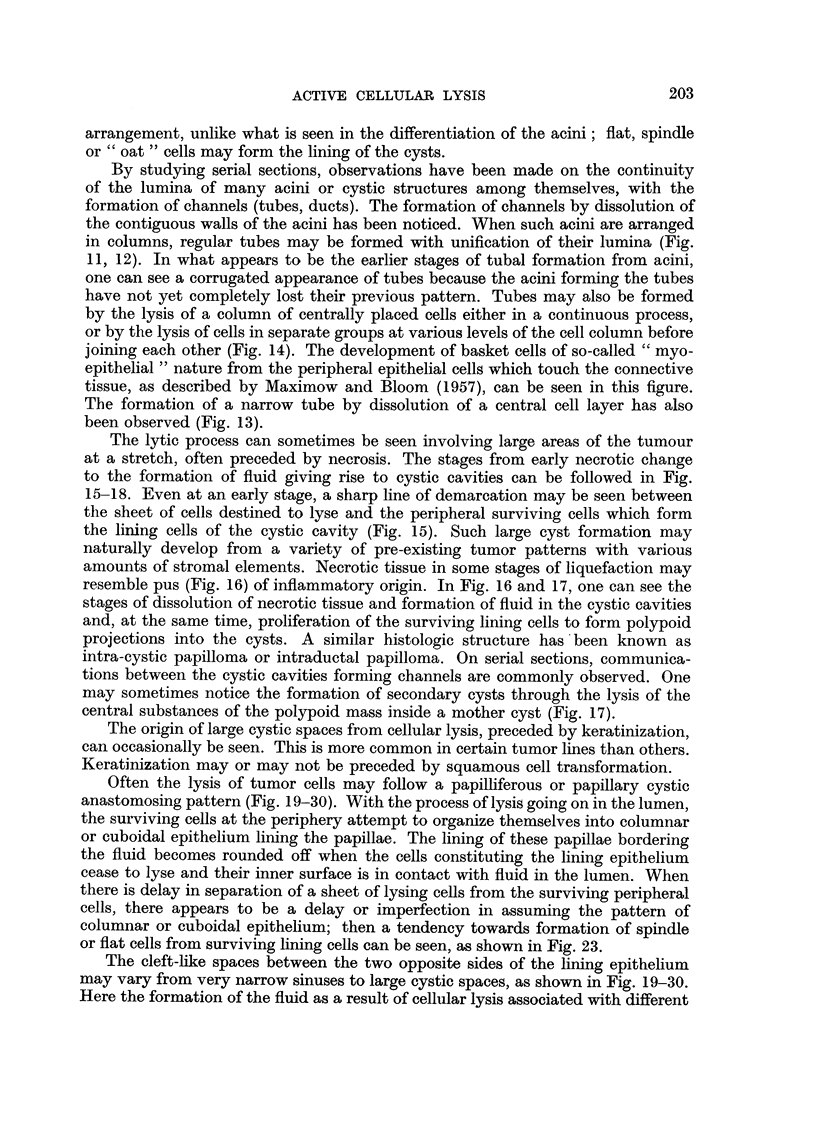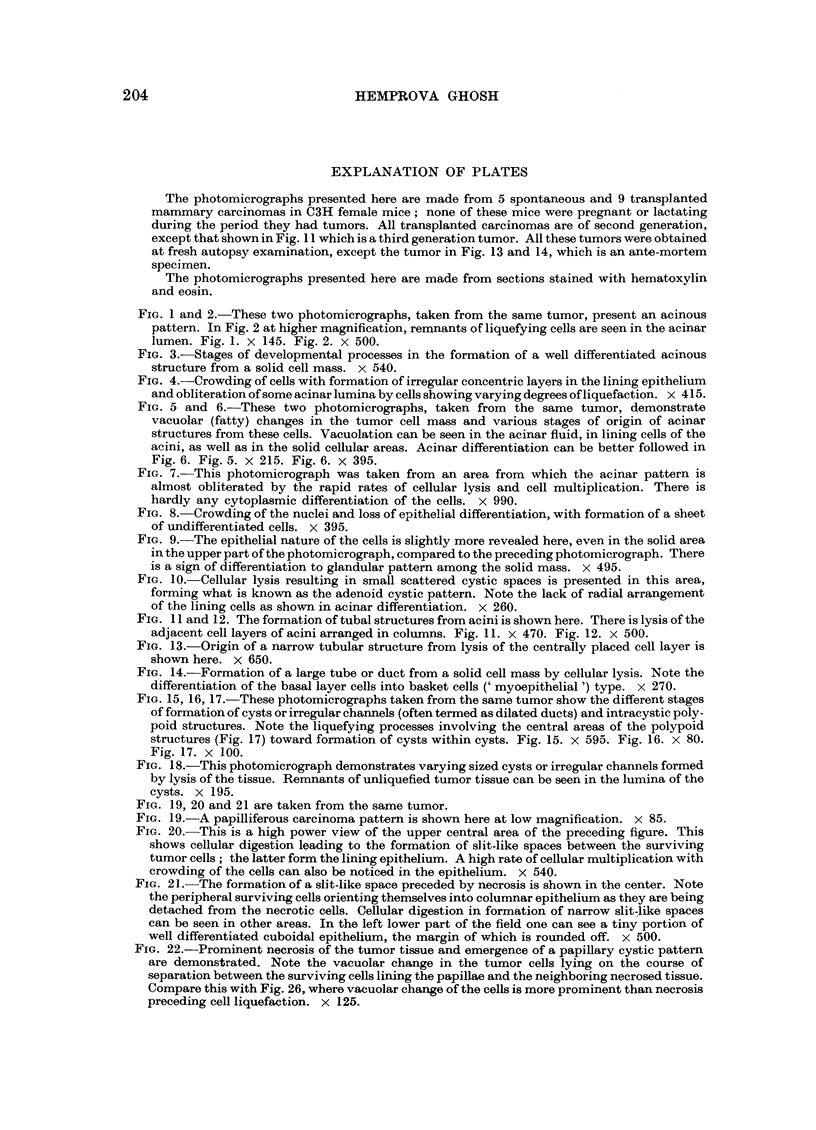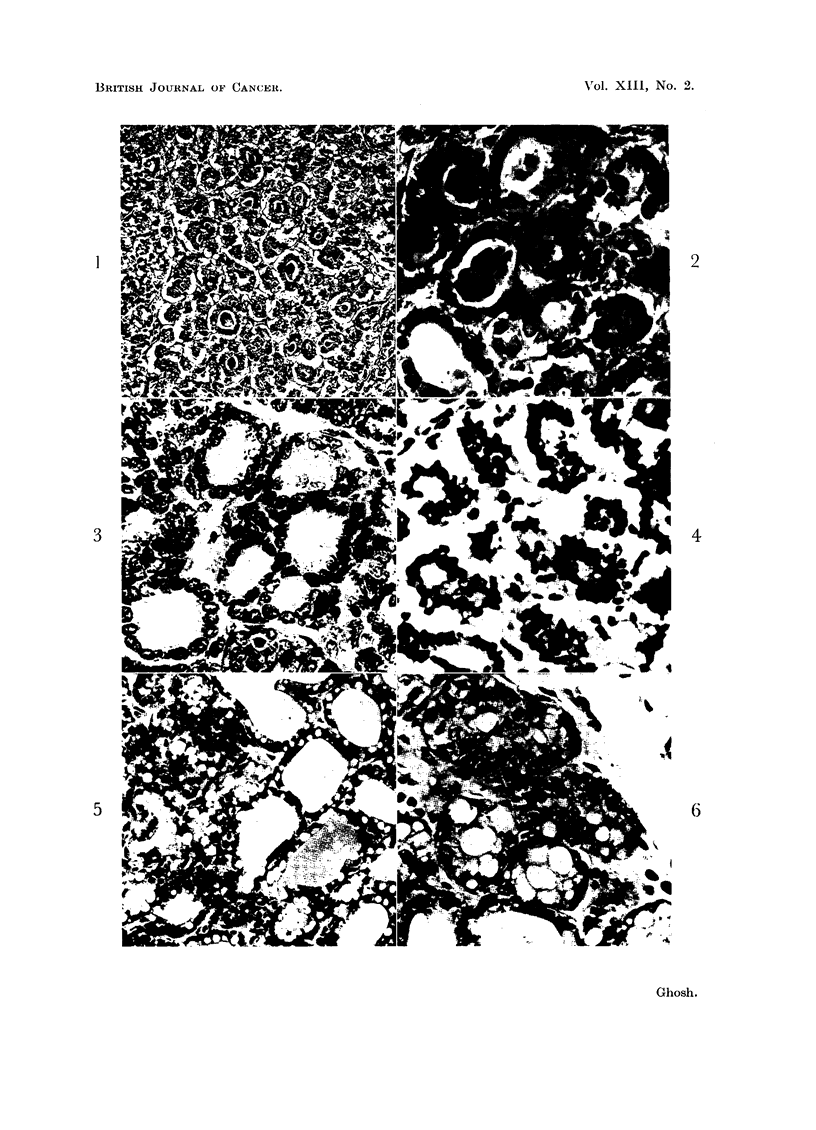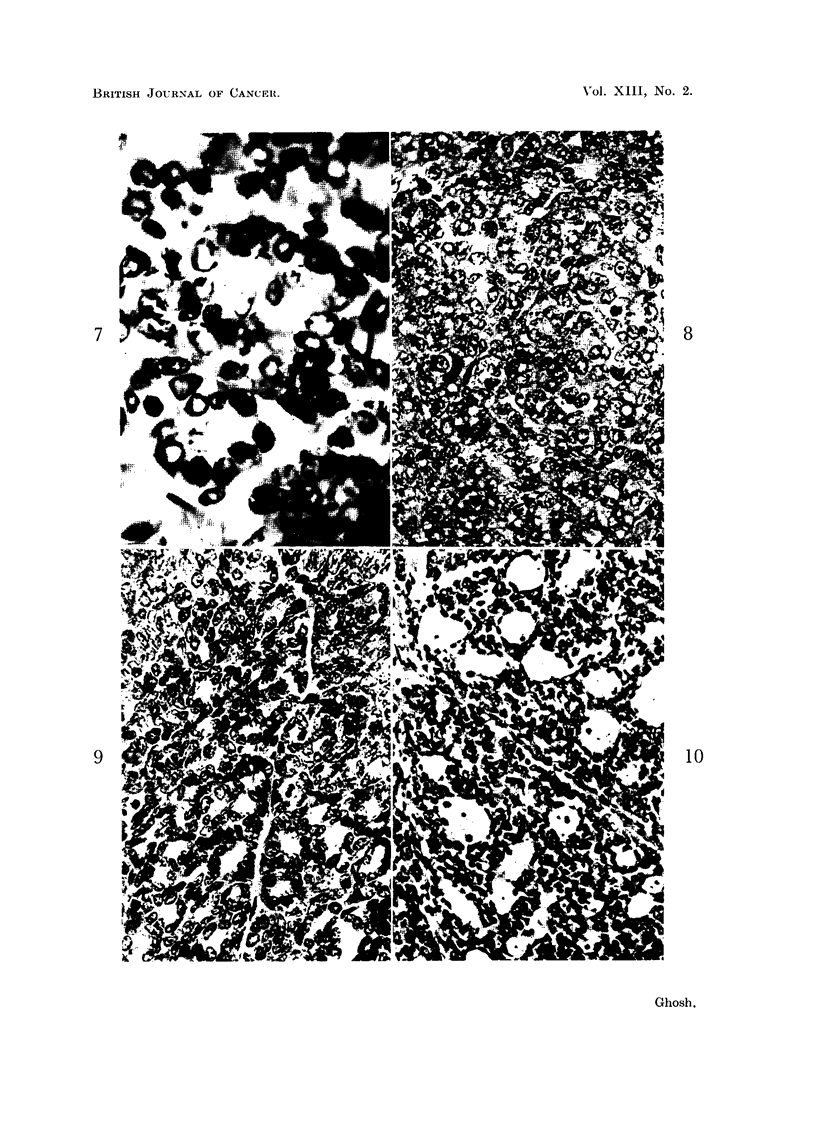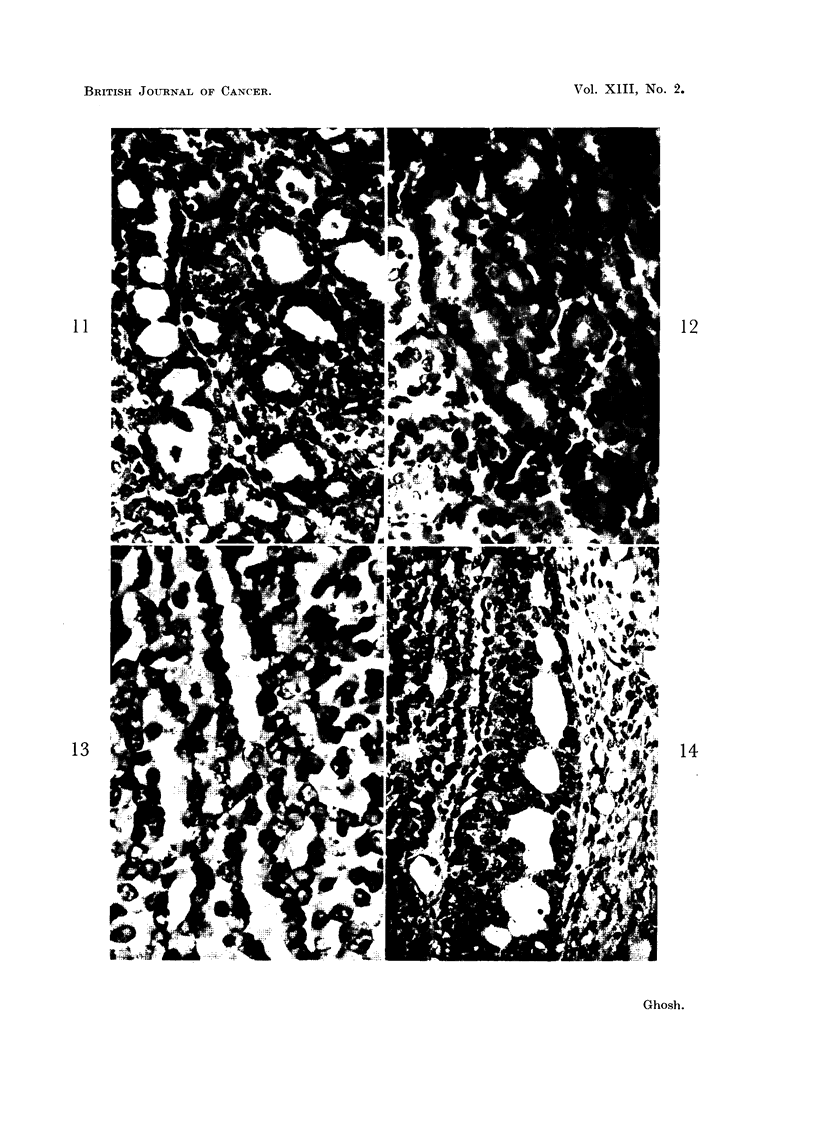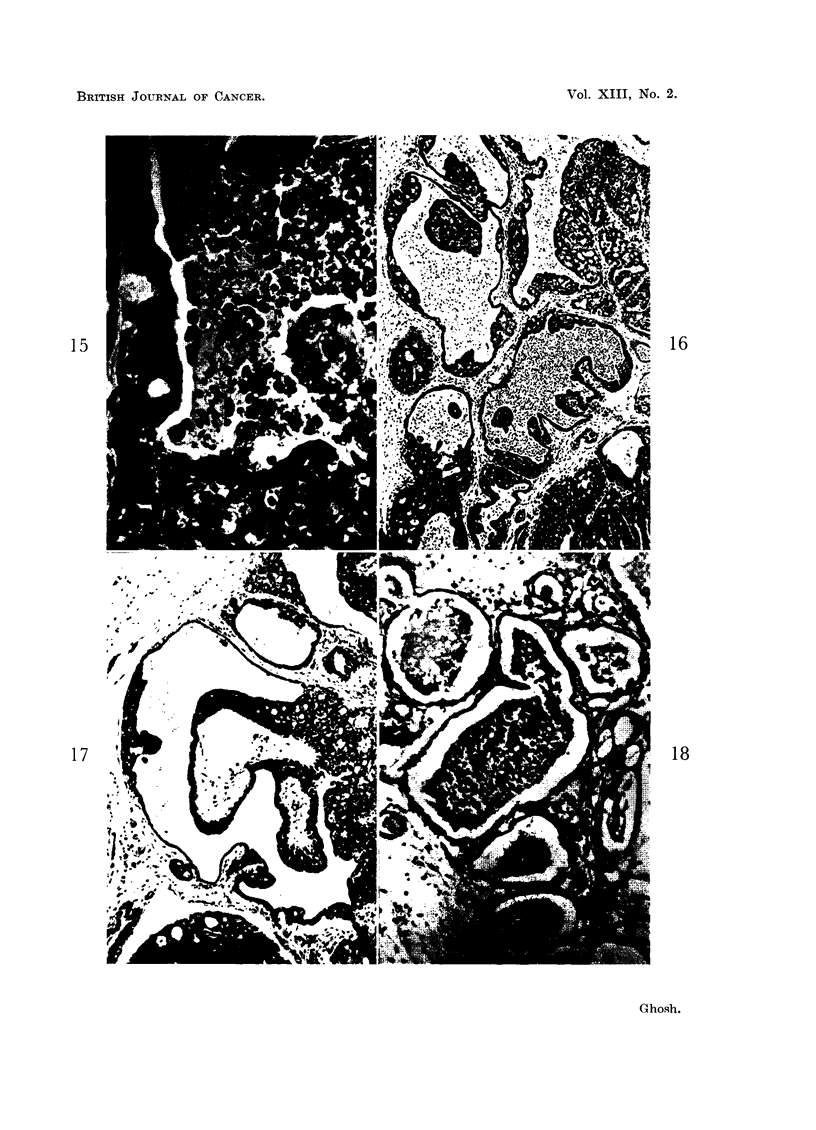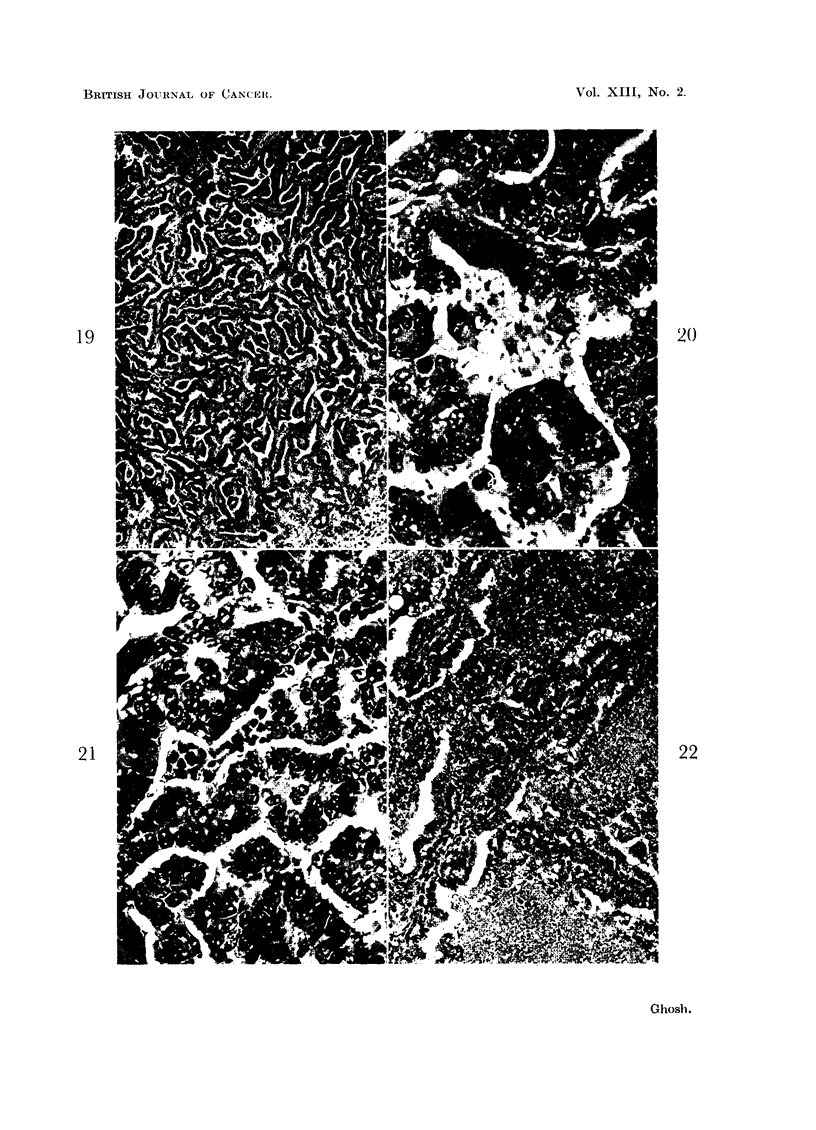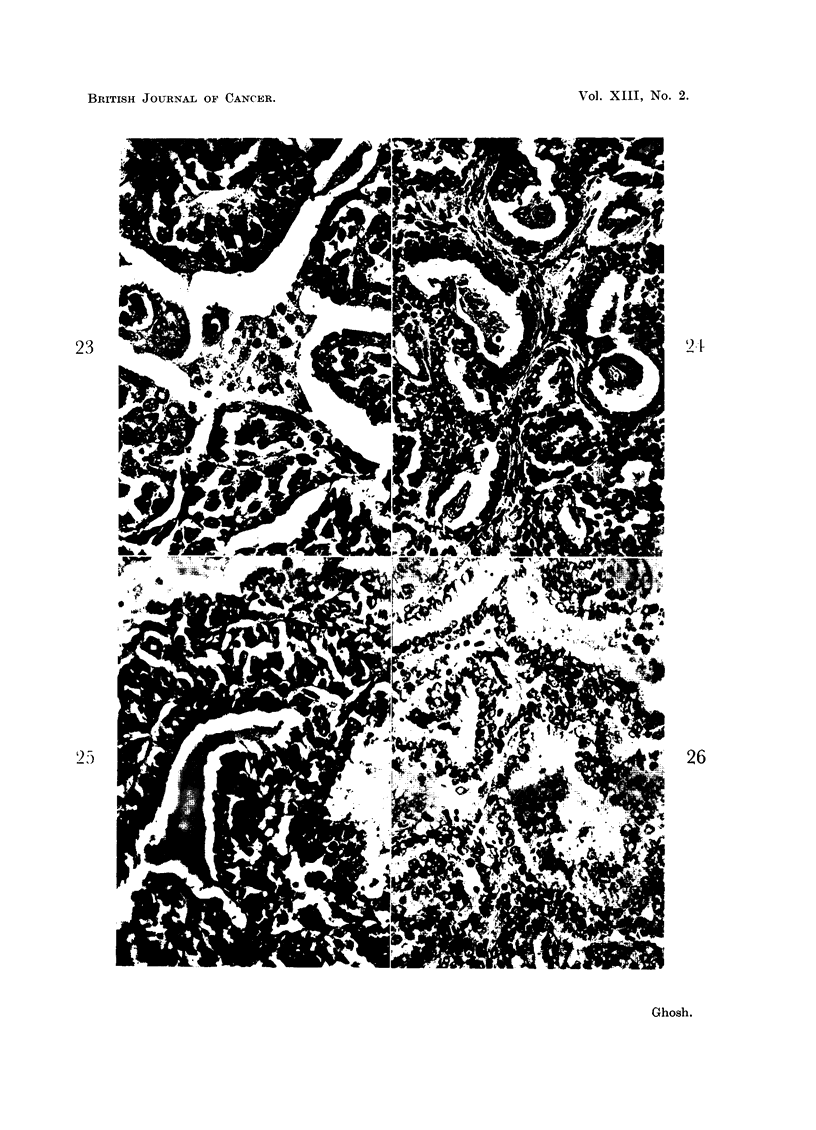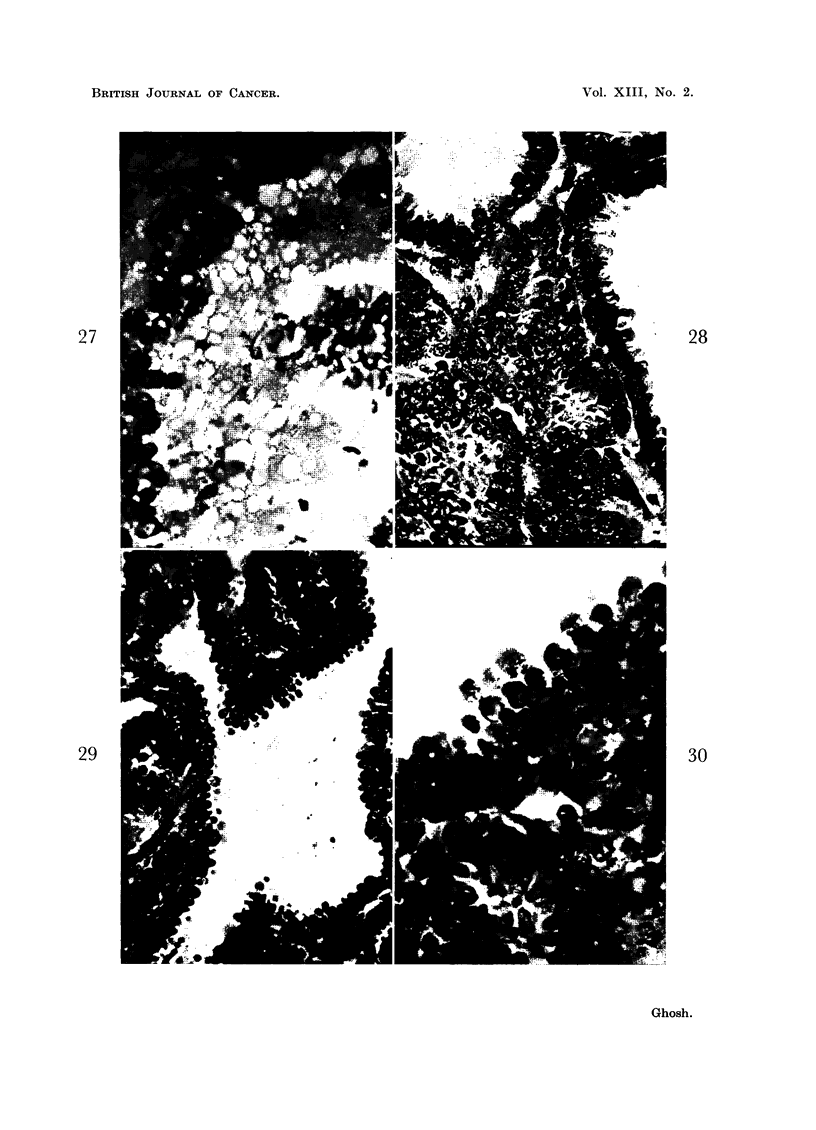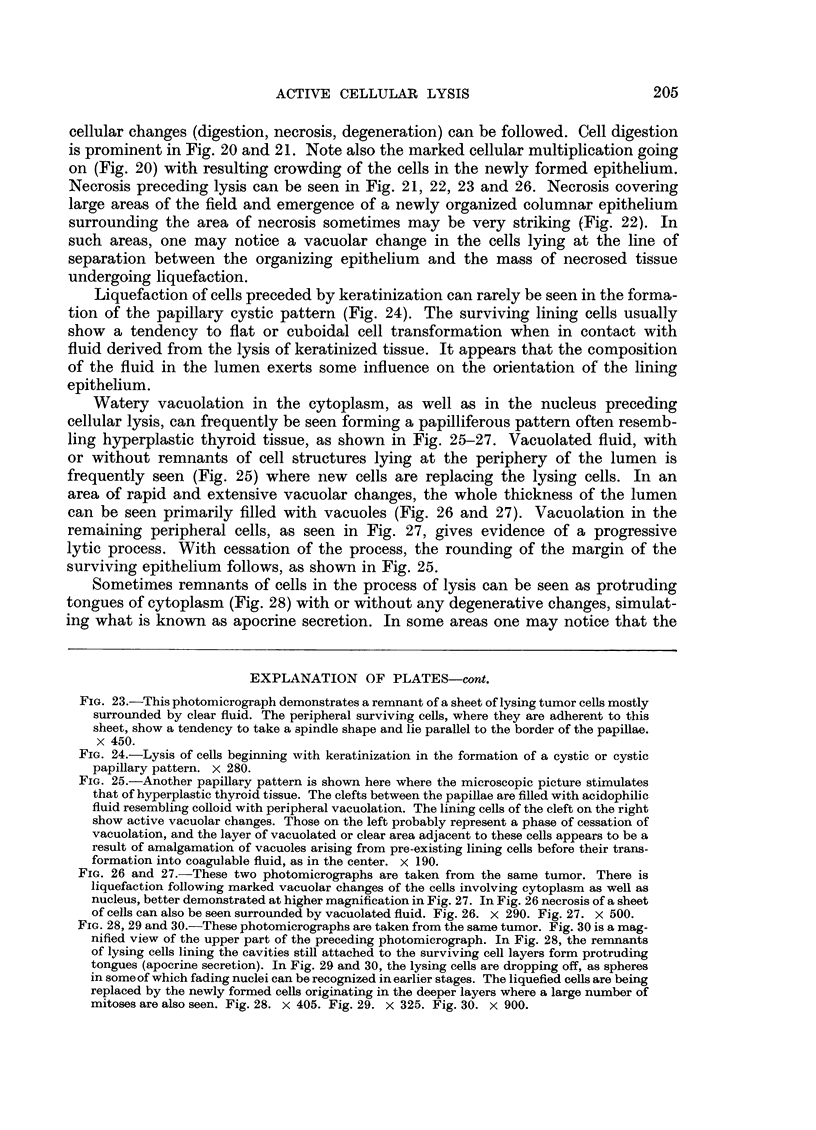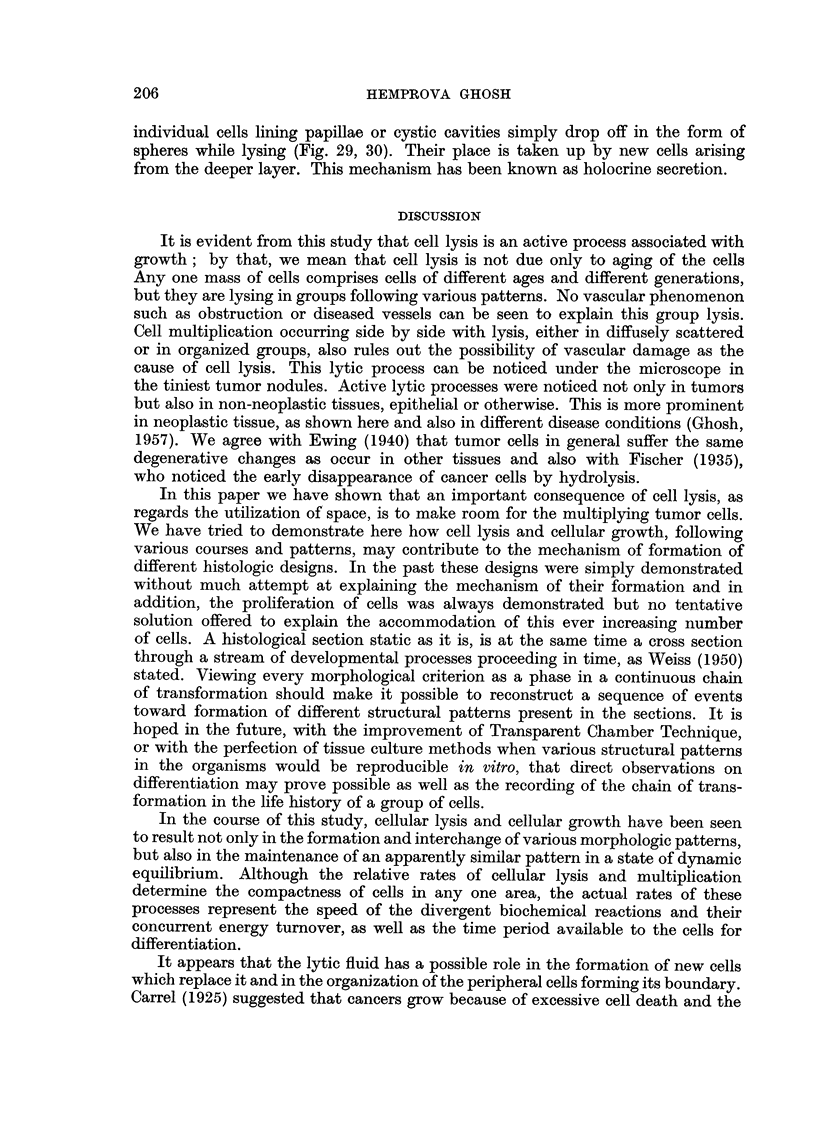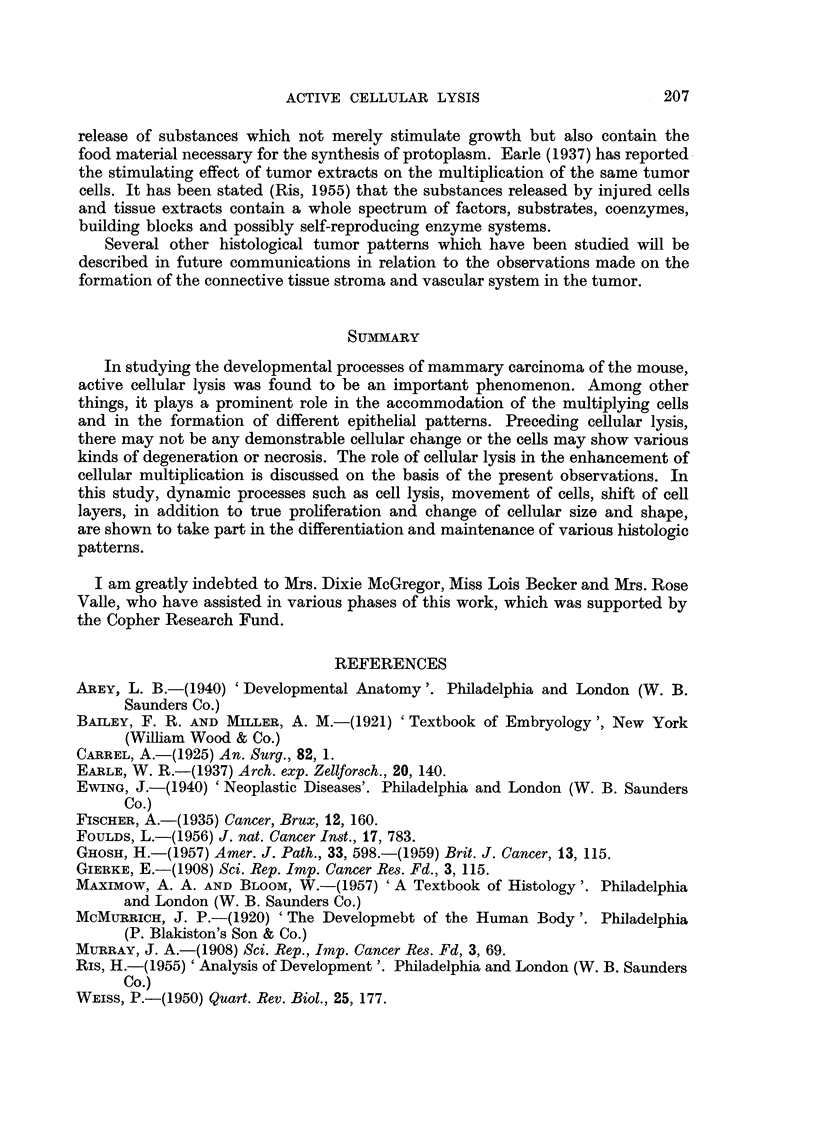# Active Cellular Lysis, a Phenomenon of Growth Processes and its Role in the Formation of Different Epithelial Patterns as shown in Mammary Carcinomas in Mice*

**DOI:** 10.1038/bjc.1959.27

**Published:** 1959-06

**Authors:** Hemprova Ghosh

## Abstract

**Images:**


### ACTIVE CELLULAR LYSIS, A PHENOMENON OF GROWTH PROCESSES AND ITS ROLE IN THE FORMATION OF DIFFERENT EPITHELIAL PATTERNS AS SHOWN IN MAMMARY CARCINOMAS IN MICE*

HEMPROVA GHOSH

*From the Department of Surgical Pathology, Washington University School of Medicine and Barnard Free Skin and Cancer Hospital, St. Louis, Missouri, U.S.A.*

It is generally assumed that the histologic pattern which the growth of a particular tumor takes is in some way determined by an intrinsic characteristic of the tumor cell. But the great influence exerted by the host tissue or the medium in which the tumor is growing in determination of its histologic pattern as well as its biological behavior, has not been denied.

In the course of the microscopical study of a large number of mammary carcinomata of mice, it was realized that the developmental processes of tumors depend not only on cellular multiplication but also on lysis (dissolution) of the tumor tissue, including both parenchyma and stroma, in varied patterns. It appears that cell dissolution is as important as cellular multiplication in formation and maintenance of the structural patterns, and the present study deals with the developmental processes of a tumor growing in its natural host.

### MATERIALS AND METHOD

The observations presented are based on the study of over 1500 mammary carcinomata in C3H female mice. This includes 1048 transplanted and 71 spontaneous tumors obtained at autopsy examination ; a part of the same material was used in a previous paper (Ghosh, 1959). It also includes biopsy specimens obtained from 44 spontaneous tumors and specimens obtained by antemortem excision of 315 transplanted carcinomas and 51 spontaneous carcinomas. A large number of the tumour-bearing animals were used in our experimental chemotherapy program. No appreciable difference was noticed between treated and untreated groups, with respect to the histologic picture of the tumor.

Tumors were fixed in 10 per cent neutral formalin and sectioned at 4 micron thickness and stained with hematoxylin and eosin for routine study. For confirmation of the shapes of certain histological structures in their entirety in three dimensions, serial sections of the tumors made at 4 to 6 micron thickness were examined. Special stains for the detection of mucin, amyloid, iron, calcium, hemoglobin, melanin, elastic tissue, reticulin, muscle, fat, PAS positive material, etc. were employed on some specimens. Both light and phase contrast microscopy were used.

* A partial presentation of this paper was made at the Fifty-fifth Annual Meeting of the American Association of Pathologists and Bacteriologists.

### OBSERVATIONS

The formation of various histological patterns in carcinoma was studied by observing the cellular changes which developed during the growth of mammary carcinomata in mice. In this paper the observations are limited to the various cellular changes associated with cell lysis and to reorientation of the surviving and newly formed cells into several histologic designs with retention of their epithelial nature.

In the course of the lytic process, which involves both epithelial and stromal elements, various intermediate stages are recognisable, which may be called digestion, degeneration and necrosis. After first noting each of the cellular changes leading to dissolution and the changing characters of the fluid, we shall illustrate the lytic phenomena, as well as the cellular growth and arrangement in the formation of various tumor patterns. By digestion, we mean simply the dissolving out of the cytoplasmic and nuclear substances, as one sees in the case of cells digested *in vitro* by various proteolytic enzymes, without any evidence of degeneration or necrosis. Cellular lysis has been seen associated with changes which fall under the category of various degenerative processes, such as hyaline, hydropic, fibrinoid, granular, vacuolar (watery or fatty), mucoid, myxoid, amyloid, keratoid, etc. The granular and vacuolar configurations have been termed the secretory stage and the lytic product, when observed within a lumen and bound by a lining epithelium has been called secretion. Cellular lysis may be preceded by necrosis. During this process, in certain stages the cellular changes may be indistinguishable from what is usually considered as pus in inflammatory conditions. Sometimes a cellular change known as aseptic necrosis may precede cell lysis. No vascular obstruction is noticed to explain the necrosis. Necrosis was seen in tumor nodules measuring less than 1 mm. in diameter. One or more than one of the cellular changes associated with lysis mentioned above may take place in a group of cells.

The product of cellular lysis (secretion) may exhibit further and various changes in its biochemical make-up, as reflected by the varied nature of its staining characteristics from one area to another : clear (vacuoles), various shades of colors, granulation, globule formation, rarely calcification and others. The changing characters of the fluid have often been termed maturation. By using different staining techniques, reactions in the fluid become even more prominent. In this paper we shall not deal with these phenomena in any detail, since only black and white prints of photomicrographs are presented and since its purpose is to draw attention to the phenomenon of cellular lysis as an active process in the growth of a tumor.

Since the normal mammary gland presents a tubo-acinar pattern primarily, the developmental processes of an acinar structure in tumors are presented first (Fig. 1–6). The formation of the acinar lumen and accumulation of fluid in it, as a result of cellular lysis involving scattered round areas, can be well followed in each of Fig. 2, 3 and 6. Here one can also notice the stages of reorientation of the surviving peripheral cells in the formation of cuboidal epithelium, which encircles the products of cell lysis. In what appears to be a quiescent stage of developmental processes including both cellular lysis and multiplication (a rapid rate of which is shown in Fig. 7) and when in contact with fluid, the inner margin of the surviving cells forming lining epithelium becomes rounded and a well differentiated single cell layer epithelium is formed.

In the process of the formation of acinar fluid from a solid cell mass, one may notice clumping of cells with hyalinization (Fig. 2) or vacuolation of the cells in the acinar lumina (Fig. 2 and 3). In Fig. 3 one can see the fading away of the cells with the appearance of watery vacuoles in the interstices of the remnants of lysing cells and formation of homogeneous coagulable fluid. Here stages of " maturation " of the fluid and organization of the lining epithelium are going on hand-in-hand toward formation of well differentiated acini. The formation of tubo acinar structures from solid cell masses in cancer was recognized by early workers (Murray, 1908 ; Gierke, 1908) and the origin of tubo acinar structures of the mammary gland from solid cell masses was observed by both histologists and embryologists (Maximow and Bloom, 1957 ; Arey, 1940 ; Bailey and Miller, 1921 ; McMurrich, 1920). They pointed out that the appearance of the lumina in these structures is caused by a separation and breaking down of the central cells of ducts and acini, the peripheral cells remaining as their lining.

In Fig. 5 and 6, which simulate lactating mammary glands, one can see cellular lysis associated with fatty vacuolation of the cytoplasm and nucleus toward formation of acinar fluid. The lysis of cells following fatty changes (secretion, degeneration) can be seen in the process of differentiation toward acini. Some cells showing fatty vacuoles can be seen in the inter-acinar areas. Foulds (1956) has pointed out the prominent milky secretion in spontaneous and transplanted mammary carcinomas of the mouse when the animals are pregnant, lactating, or receive estrogens. He also stated, " Occasionally it is questionable whether the accumulation of fat should be ascribed to secretion or degeneration."

In an area of a well differentiated epithelial pattern (acinar or other types) with a single layer of lining cells with more or less rounded margin, the rates of cellular lysis and formation of new replacement cells appear to be slow and balance each other.

It is possible that when the lytic process in the acinar cells is slow compared to cellular multiplication, the acinar pattern is gradually obliterated. In such acini, concentric layers or crowding of lining cells may be found because of the persistence of inner layer cells due to delay in lysis, as shown in Fig. 4. The enlargement of the lumen (often called dilatation) of a glandular structure appears to be due to the peripheral extension of the area of cellular lysis and to the new epithelium forming at an outer zone, instead of replacing the previous one *in situ*.

In some areas a high rate of cellular multiplication can be seen in the acinar and inter-acinar cells, associated with rapid cellular lysis, where there is hardly any cytoplasmic dfferentiation (Fig. 7). It is conceivable that with a relative slowing down of the speed of cellular lysis, this can be transformed into a solid sheet of poorly differentiated cells, as shown in Fig. 8, in which crowded nuclei can be seen lying in a pool of cytoplasm. On close examination, many scattered nuclei can be seen in various stages of lysis. This stage may be followed by a more quiescent stage, where the speed of cellular multiplication is slow. In such areas (Fig. 9) one may notice the attempts at differentiation into a glandular pattern from a solid sheet of tumor cells. Both Murray (1908) and Gierke (1908) have pointed this out.

The lytic processes sometimes lead to the formation of scattered small cysts, as seen in the formation of the adenoid cystic pattern (Fig. 10). Myxoid, mucoid, hyaline, hydropic or fibrinoid changes are usually associated with cellular lysis in the origin of the adenoid cystic pattern. The surviving lining cells lack radial arrangement, unlike what is seen in the differentiation of the acini ; flat, spindle or " oat " cells may form the lining of the cysts.

By studying serial sections, observations have been made on the continuity of the lumina of many acini or cystic structures among themselves, with the formation of channels (tubes, ducts). The formation of channels by dissolution of the contiguous walls of the acini has been noticed. When such acini are arranged in columns, regular tubes may be formed with unification of their lumina (Fig. 11, 12). In what appears to be the earlier stages of tubal formation from acini, one can see a corrugated appearance of tubes because the acini forming the tubes have not yet completely lost their previous pattern. Tubes may also be formed by the lysis of a column of centrally placed cells either in a continuous process, or by the lysis of cells in separate groups at various levels of the cell column before joining each other (Fig. 14). The development of basket cells of so-called " myo-epithelial " nature from the peripheral epithelial cells which touch the connective tissue, as described by Maximow and Bloom (1957), can be seen in this figure. The formation of a narrow tube by dissolution of a central cell layer has also been observed (Fig. 13).

The lytic process can sometimes be seen involving large areas of the tumour at a stretch, often preceded by necrosis. The stages from early necrotic change to the formation of fluid giving rise to cystic cavities can be followed in Fig. 15–18. Even at an early stage, a sharp line of demarcation may be seen between the sheet of cells destined to lyse and the peripheral surviving cells which form the lining cells of the cystic cavity (Fig. 15). Such large cyst formation may naturally develop from a variety of pre-existing tumor patterns with various amounts of stromal elements. Necrotic tissue in some stages of liquefaction may resemble pus (Fig. 16) of inflammatory origin. In Fig. 16 and 17, one can see the stages of dissolution of necrotic tissue and formation of fluid in the cystic cavities and, at the same time, proliferation of the surviving lining cells to form polypoid projections into the cysts. A similar histologic structure has been known as intra-cystic papilloma or intraductal papilloma. On serial sections, communications between the cystic cavities forming channels are commonly observed. One may sometimes notice the formation of secondary cysts through the lysis of the central substances of the polypoid mass inside a mother cyst (Fig. 17).

The origin of large cystic spaces from cellular lysis, preceded by keratinization, can occasionally be seen. This is more common in certain tumor lines than others. Keratinization may or may not be preceded by squamous cell transformation.

Often the lysis of tumor cells may follow a papilliferous or papillary cystic anastomosing pattern (Fig. 19–30). With the process of lysis going on in the lumen, the surviving cells at the periphery attempt to organize themselves into columnar or cuboidal epithelium lining the papillae. The lining of these papillae bordering the fluid becomes rounded off when the cells constituting the lining epithelium cease to lyse and their inner surface is in contact with fluid in the lumen. When there is delay in separation of a sheet of lysing cells from the surviving peripheral cells, there appears to be a delay or imperfection in assuming the pattern of columnar or cuboidal epithelium; then a tendency towards formation of spindle or flat cells from surviving lining cells can be seen, as shown in Fig. 23.

The cleft-like spaces between the two opposite sides of the lining epithelium may vary from very narrow sinuses to large cystic spaces, as shown in Fig. 19–30. Here the formation of the fluid as a result of cellular lysis associated with different

### EXPLANATION OF PLATES

The photomicrographs presented here are made from 5 spontaneous and 9 transplanted mammary carcinomas in C3H female mice ; none of these mice were pregnant or lactating during the period they had tumors. All transplanted carcinomas are of second generation, except that shown in Fig. 11 which is a third generation tumor. All these tumors were obtained at fresh autopsy examination, except the tumor in Fig. 13 and 14, which is an ante-mortem specimen.

The photomicrographs presented here are made from sections stained with hematoxylin and eosin.

FIG. 1 and 2.—These two photomicrographs, taken from the same tumor, present an acinous pattern. In Fig. 2 at higher magnification, remnants of liquefying cells are seen in the acinar lumen. Fig. 1. × 145. Fig. 2. × 500.

FIG. 3.—Stages of developmental processes in the formation of a well differentiated acinous structure from a solid cell mass. × 540.

FIG. 4.—Crowding of cells with formation of irregular concentric layers in the lining epithelium and obliteration of some acinar lumina by cells showing varying degrees of liquefaction. × 415.

FIG. 5 and 6.—These two photomicrographs, taken from the same tumor, demonstrate vacuolar (fatty) changes in the tumor cell mass and various stages of origin of acinar structures from these cells. Vacuolation can be seen in the acinar fluid, in lining cells of the acini, as well as in the solid cellular areas. Acinar differentiation can be better followed in Fig. 6. Fig. 5. × 215. Fig. 6. × 395.

FIG. 7.—This photomicrograph was taken from an area from which the acinar pattern is almost obliterated by the rapid rates of cellular lysis and cell multiplication. There is hardly any cytoplasmic differentiation of the cells. × 990.

FIG. 8.—Crowding of the nuclei and loss of epithelial differentiation, with formation of a sheet of undifferentiated cells. × 395.

FIG. 9.—The epithelial nature of the cells is slightly more revealed here, even in the solid area in the upper part of the photomicrograph, compared to the preceding photomicrograph. There is a sign of differentiation to glandular pattern among the solid mass. × 495.

FIG. 10.—Cellular lysis resulting in small scattered cystic spaces is presented in this area, forming what is known as the adenoid cystic pattern. Note the lack of radial arrangement of the lining cells as shown in acinar differentiation. × 260.

FIG. 11 and 12. The formation of tubal structures from acini is shown here. There is lysis of the adjacent cell layers of acini arranged in columns. Fig. 11. × 470. Fig. 12. × 500.

FIG. 13.—Origin of a narrow tubular structure from lysis of the centrally placed cell layer is shown here. × 650.

FIG. 14.—Formation of a large tube or duct from a solid cell mass by cellular lysis. Note the differentiation of the basal layer cells into basket cells (' myoepithelial ') type. × 270.

FIG. 15, 16, 17.—These photomicrographs taken from the same tumor show the different stages of formation of cysts or irregular channels (often termed as dilated ducts) and intracystic polypoid structures. Note the liquefying processes involving the central areas of the polypoid structures (Fig. 17) toward formation of cysts within cysts. Fig. 15. × 595. Fig. 16. × 80. Fig. 17. × 100.

FIG. 18.—This photomicrograph demonstrates varying sized cysts or irregular channels formed by lysis of the tissue. Remnants of unliquefied tumor tissue can be seen in the lumina of the cysts. × 195.

FIG. 19, 20 and 21 are taken from the same tumor.

FIG. 19.—A papilliferous carcinoma pattern is shown here at low magnification. × 85.

FIG. 20.—This is a high power view of the upper central area of the preceding figure. This shows cellular digestion leading to the formation of slit-like spaces between the surviving tumor cells ; the latter form the lining epithelium. A high rate of cellular multiplication with crowding of the cells can also be noticed in the epithelium. × 540.

FIG. 21.—The formation of a slit-like space preceded by necrosis is shown in the center. Note the peripheral surviving cells orienting themselves into columnar epithelium as they are being detached from the necrotic cells. Cellular digestion in formation of narrow slit-like spaces can be seen in other areas. In the left lower part of the field one can see a tiny portion of well differentiated cuboidal epithelium, the margin of which is rounded off. × 500.

FIG. 22.—Prominent necrosis of the tumor tissue and emergence of a papillary cystic pattern are demonstrated. Note the vacuolar change in the tumor cells lying on the course of separation between the surviving cells lining the papillae and the neighboring necrosed tissue. Compare this with Fig. 26, where vacuolar change of the cells is more prominent than necrosis preceding cell liquefaction. × 125.

1 2

3 4

5 6

Ghosh.

7

8

9

10

11

12

13

14

Ghosh.

15

16

17

18

19 20

21 22

23

24

25

26

27

28

29

30

cellular changes (digestion, necrosis, degeneration) can be followed. Cell digestion is prominent in Fig. 20 and 21. Note also the marked cellular multiplication going on (Fig. 20) with resulting crowding of the cells in the newly formed epithelium. Necrosis preceding lysis can be seen in Fig. 21, 22, 23 and 26. Necrosis covering large areas of the field and emergence of a newly organized columnar epithelium surrounding the area of necrosis sometimes may be very striking (Fig. 22). In such areas, one may notice a vacuolar change in the cells lying at the line of separation between the organizing epithelium and the mass of necrosed tissue undergoing liquefaction.

Liquefaction of cells preceded by keratinization can rarely be seen in the formation of the papillary cystic pattern (Fig. 24). The surviving lining cells usually show a tendency to flat or cuboidal cell transformation when in contact with fluid derived from the lysis of keratinized tissue. It appears that the composition of the fluid in the lumen exerts some influence on the orientation of the lining epithelium.

Watery vacuolation in the cytoplasm, as well as in the nucleus preceding cellular lysis, can frequently be seen forming a papilliferous pattern often resembling hyperplastic thyroid tissue, as shown in Fig. 25–27. Vacuolated fluid, with or without remnants of cell structures lying at the periphery of the lumen is frequently seen (Fig. 25) where new cells are replacing the lysing cells. In an area of rapid and extensive vacuolar changes, the whole thickness of the lumen can be seen primarily filled with vacuoles (Fig. 26 and 27). Vacuolation in the remaining peripheral cells, as seen in Fig. 27, gives evidence of a progressive lytic process. With cessation of the process, the rounding of the margin of the surviving epithelium follows, as shown in Fig. 25.

Sometimes remnants of cells in the process of lysis can be seen as protruding tongues of cytoplasm (Fig. 28) with or without any degenerative changes, simulating what is known as apocrine secretion. In some areas one may notice that the

---

EXPLANATION OF PLATES—*cont.*

FIG. 23.—This photomicrograph demonstrates a remnant of a sheet of lysing tumor cells mostly surrounded by clear fluid. The peripheral surviving cells, where they are adherent to this sheet, show a tendency to take a spindle shape and lie parallel to the border of the papillae. × 450.

FIG. 24.—Lysis of cells beginning with keratinization in the formation of a cystic or cystic papillary pattern. × 280.

FIG. 25.—Another papillary pattern is shown here where the microscopic picture stimulates that of hyperplastic thyroid tissue. The clefts between the papillae are filled with acidophilic fluid resembling colloid with peripheral vacuolation. The lining cells of the cleft on the right show active vacuolar changes. Those on the left probably represent a phase of cessation of vacuolation, and the layer of vacuolated or clear area adjacent to these cells appears to be a result of amalgamation of vacuoles arising from pre-existing lining cells before their transformation into coagulable fluid, as in the center. × 190.

FIG. 26 and 27.—These two photomicrographs are taken from the same tumor. There is liquefaction following marked vacuolar changes of the cells involving cytoplasm as well as nucleus, better demonstrated at higher magnification in Fig. 27. In Fig. 26 necrosis of a sheet of cells can also be seen surrounded by vacuolated fluid. Fig. 26. × 290. Fig. 27. × 500.

FIG. 28, 29 and 30.—These photomicrographs are taken from the same tumor. Fig. 30 is a magnified view of the upper part of the preceding photomicrograph. In Fig. 28, the remnants of lysing cells lining the cavities still attached to the surviving cell layers form protruding tongues (apocrine secretion). In Fig. 29 and 30, the lysing cells are dropping off, as spheres in some of which fading nuclei can be recognized in earlier stages. The liquefied cells are being replaced by the newly formed cells originating in the deeper layers where a large number of mitoses are also seen. Fig. 28. × 405. Fig. 29. × 325. Fig. 30. × 900.

individual cells lining papillae or cystic cavities simply drop off in the form of spheres while lysing (Fig. 29, 30). Their place is taken up by new cells arising from the deeper layer. This mechanism has been known as holocrine secretion.

### DISCUSSION

It is evident from this study that cell lysis is an active process associated with growth ; by that, we mean that cell lysis is not due only to aging of the cells Any one mass of cells comprises cells of different ages and different generations, but they are lysing in groups following various patterns. No vascular phenomenon such as obstruction or diseased vessels can be seen to explain this group lysis. Cell multiplication occurring side by side with lysis, either in diffusely scattered or in organized groups, also rules out the possibility of vascular damage as the cause of cell lysis. This lytic process can be noticed under the microscope in the tiniest tumor nodules. Active lytic processes were noticed not only in tumors but also in non-neoplastic tissues, epithelial or otherwise. This is more prominent in neoplastic tissue, as shown here and also in different disease conditions (Ghosh, 1957). We agree with Ewing (1940) that tumor cells in general suffer the same degenerative changes as occur in other tissues and also with Fischer (1935), who noticed the early disappearance of cancer cells by hydrolysis.

In this paper we have shown that an important consequence of cell lysis, as regards the utilization of space, is to make room for the multiplying tumor cells. We have tried to demonstrate here how cell lysis and cellular growth, following various courses and patterns, may contribute to the mechanism of formation of different histologic designs. In the past these designs were simply demonstrated without much attempt at explaining the mechanism of their formation and in addition, the proliferation of cells was always demonstrated but no tentative solution offered to explain the accommodation of this ever increasing number of cells. A histological section static as it is, is at the same time a cross section through a stream of developmental processes proceeding in time, as Weiss (1950) stated. Viewing every morphological criterion as a phase in a continuous chain of transformation should make it possible to reconstruct a sequence of events toward formation of different structural patterns present in the sections. It is hoped in the future, with the improvement of Transparent Chamber Technique, or with the perfection of tissue culture methods when various structural patterns in the organisms would be reproducible *in vitro*, that direct observations on differentiation may prove possible as well as the recording of the chain of transformation in the life history of a group of cells.

In the course of this study, cellular lysis and cellular growth have been seen to result not only in the formation and interchange of various morphologic patterns, but also in the maintenance of an apparently similar pattern in a state of dynamic equilibrium. Although the relative rates of cellular lysis and multiplication determine the compactness of cells in any one area, the actual rates of these processes represent the speed of the divergent biochemical reactions and their concurrent energy turnover, as well as the time period available to the cells for differentiation.

It appears that the lytic fluid has a possible role in the formation of new cells which replace it and in the organization of the peripheral cells forming its boundary. Carrel (1925) suggested that cancers grow because of excessive cell death and the release of substances which not merely stimulate growth but also contain the food material necessary for the synthesis of protoplasm. Earle (1937) has reported the stimulating effect of tumor extracts on the multiplication of the same tumor cells. It has been stated (Ris, 1955) that the substances released by injured cells and tissue extracts contain a whole spectrum of factors, substrates, coenzymes, building blocks and possibly self-reproducing enzyme systems.

Several other histological tumor patterns which have been studied will be described in future communications in relation to the observations made on the formation of the connective tissue stroma and vascular system in the tumor.

### Summary

In studying the developmental processes of mammary carcinoma of the mouse, active cellular lysis was found to be an important phenomenon. Among other things, it plays a prominent role in the accommodation of the multiplying cells and in the formation of different epithelial patterns. Preceding cellular lysis, there may not be any demonstrable cellular change or the cells may show various kinds of degeneration or necrosis. The role of cellular lysis in the enhancement of cellular multiplication is discussed on the basis of the present observations. In this study, dynamic processes such as cell lysis, movement of cells, shift of cell layers, in addition to true proliferation and change of cellular size and shape, are shown to take part in the differentiation and maintenance of various histologic patterns.

I am greatly indebted to Mrs. Dixie McGregor, Miss Lois Becker and Mrs. Rose Valle, who have assisted in various phases of this work, which was supported by the Copher Research Fund.

### REFERENCES

Arey, L. B.—(1940) 'Developmental Anatomy'. Philadelphia and London (W. B. Saunders Co.)

Bailey, F. R. and Miller, A. M.—(1921) 'Textbook of Embryology', New York (William Wood & Co.)

Carrel, A.—(1925) *An. Surg.*, **82**, 1.

Earle, W. R.—(1937) *Arch. exp. Zellforsch.*, **20**, 140.

Ewing, J.—(1940) 'Neoplastic Diseases'. Philadelphia and London (W. B. Saunders Co.)

Fischer, A.—(1935) *Cancer, Brux*, **12**, 160.

Foulds, L.—(1956) *J. nat. Cancer Inst.*, **17**, 783.

Ghosh, H.—(1957) *Amer. J. Path.*, **33**, 598.—(1959) *Brit. J. Cancer*, **13**, 115.

Gierke, E.—(1908) *Sci. Rep. Imp. Cancer Res. Fd.*, **3**, 115.

Maximow, A. A. and Bloom, W.—(1957) 'A Textbook of Histology'. Philadelphia and London (W. B. Saunders Co.)

McMurrich, J. P.—(1920) 'The Developmebt of the Human Body'. Philadelphia (P. Blakiston's Son & Co.)

Murray, J. A.—(1908) *Sci. Rep., Imp. Cancer Res. Fd*, **3**, 69.

Ris, H.—(1955) 'Analysis of Development'. Philadelphia and London (W. B. Saunders Co.)

Weiss, P.—(1950) *Quart. Rev. Biol.*, **25**, 177.